# Evoked Response Audiometry according to gender and age: findings and usefulness

**DOI:** 10.1016/S1808-8694(15)30601-7

**Published:** 2015-10-18

**Authors:** Edmir Américo Lourenço, Marcelo Henrique de Oliveira, Adriana Umemura, Ana Laura Vargas, Karen de Carvalho Lopes, Álvaro Vitorino de Pontes Júnior

**Affiliations:** 1 MSc in ENT-HNS and PhD in Medicine - UNIFESP, Adjunct Professor, Head of the ENT Program - FMJ.; 2 Faculdade de Medicina de Jundiaí (FMJ) - SP, ENT working in Belo Horizonte-MG.; 3 MD. Former ENT resident - Faculdade de Medicina de Jundiaí (FMJ) - SP, ENT working in Jundiaí.; 4 MD. Former ENT resident - Faculdade de Medicina de Jundiaí (FMJ) - SP, ENT working in Itu-SP.; 5 MD. Former ENT resident - Faculdade de Medicina de Jundiaí (FMJ) - SP, ENT working in Jundiaí at ATEAL- Assoc. Terap. Estim. Auditiva e Linguagem.; 6 MD. Former ENT resident - Faculdade de Medicina de Jundiaí (FMJ) - SP, ENT working in Jundiaí na ATEAL - Assoc. Terap. Estimul. Audit. e Linguagem. Faculdade de Medicina de Jundiaí - SP.

**Keywords:** findings, evoked response audiometry, age and gender distribution, auditory evoked potentials

## Abstract

Auditory evoked brainstem responses (ABR) is a non-invasive electrical potential registration which evaluates the auditory tract from the middle ear to the auditory cortex in the first 12 milliseconds (ms). The ABR is an important otoneurological evaluation.

**Aim:**

confirm the test's usefulness, major incidence and topography according to are range gender considering the absolute latencies of the waves and interpeak intervals.

**Materials and Method:**

we retrospectively analyzed 403 tests from a private clinic in the city of Jundiaí-São Paulo State-Brazil, from patients suspected of auditory damage or central nervous disorder, and the patients were broken down according to gender and age.

**Results and Conclusions:**

ABR is an important test to determinate the soundness of the auditory tract, the electrophysiological thresholds and topodiagnosis. We found no differences between type of loss and gender; there was a major incidence of retrocochlear findings among male patients between 12-20 years old; children under one year with risk factors did not present higher incidences of auditory findings when compared with all the population analyzed. The absolute latencies of waves I, III and V were higher in males, but the interpeak intervals were similar in both genders, showing that interval I-III was more frequently altered.

## INTRODUCTION

Every sensorineural structure, when submitted to a stimulus, emits bioelectrical potentials as response. Thus, the acoustic stimulation of the human auditory receptor triggers a number of electrical responses, or evoked potentials, which result in the successive activation of the cochlea and the neurons which make up the auditory pathway.^1^

The technical capacity to record electrical potentials in different levels of the nervous system in response to acoustic stimulation has produced a large number of relevant applications to otolaryngologists, audiologists and neurologists. These potentials may be recorded by non-invasive techniques, without bringing discomfort to the patient, and often without the need for sedation or anesthesia, which has increased its clinical applicability.^2^ Auditory Evoked Potentials (AEP) enable the assessment of the auditory pathway functional integrity, from the peripheral receptor organ, all the way to the cerebral cortex.

There are many expressions found in the literature, such as Brain Stem Audiometry - BERA (Brainstem Evoked Responses Audiometry), very much used in Brazil, and also the less proper expressions and which are farther apart from its properties.

ABR (Audiometry Brainstem Responses) is based on the recording and analysis of the electrophysiological activity of the peripheral auditory system all the way to the brain stem. These are early auditory evoked potentials, because the responses arise in the first 12 milliseconds (ms) after sound stimuli and it is made up of a seven wave polyphasic potential.^2^ Waves I, III and V are the most prominent, and for this reason these are the waves considered in the analysis of the plotted graph. The parameters assessed are: the very presence of the waves, replication or reproducibility, absolute latency, latency interval between waves (interpeak intervals), interaural latency difference, sound intensity, latency and amplitude.^3^

AEPs, besides enabling one to investigate the individual's peripheral hearing, they also help assess the central auditory pathway integrity, its maintenance during the development process and dysfunctions caused by many diseases.^4^

ABR's clinical applications can be divided into neurologic and audiologic, with the main goal of identifying abnormalities in the auditory nerve and the brain stem, and estimate the electrophysiological auditory threshold, based on the presence of responses to different levels of stimulus intensity. In the literature we find papers that use ABR as an auditory screening test for high risk newborns.^5^, ^6^ It is also used for intraoperative monitoring during ponscerebellar angle surgery.^7^ One of the most studied applications of this type of test is to diagnose vestibular nerve schwannomas^3^; however, there are controversies regarding the test's sensitivity and specificity.^8^

Today, ABR is very important in otoneurological assessments, and it is used by many medical specialties because of its high sensitivity, speed, objectivity and also because it enables one to follow disease progression and that of treatment.

Our goal with the present investigation is to analyze and compare ABR traces in regards to: gender distribution; test applicability to the different age ranges; risk factors correlation in newborns up to one year of age and test alterations; incidence of conductive/cochlear alterations; retrocochlear alterations; increase in wave absolute latency and interpeak interval alterations, including a differential study of these parameters in so far as gender is concerned.

## MATERIALS AND METHODS

We carried out a retrospective study, properly approved by the Ethics in Research Committee (CEP) of the Faculdade de Medicina de Jundiaí (FMJ) - SP, in a population of 403 patients who were referred to us by pediatricians, neuro-pediatricians, neurologists, neurosurgeons and otorhinolaryngologists, with doubts regarding their hearing and/or alterations in the central auditory pathways (retrocochlear), submitted to the brainstem audiometry test (ABR) in a private clinic of Jundiaí, in the State of São Paulo. The tests were carried out from June of 1998 to May of 2005 and sequentially analyzed, not excluding any chart. We used a GSI®-55 ABR Screener device, at 2000 clicks per minute, lasting 100μs (encompassing the frequencies from 2000 to 4000Hz), with one channel insertion phones and a 12 millisecond window. The sample was initially broken down by gender. The patients were broken down by age range (less than one year, between one year and less than 6 years, between 6 years and less than 12 years, between twelve years and less than 20 years, between 20 years and less than forty years, between forty years and less than 70 years, and finally, those with more than 70 years of age. As far as applicability is concerned, the tests were plotted in accordance to their end or indication (hearing threshold, retrocochlear assessment or both). As conductive and/or cochlear alterations we considered: absolute latency increase of all the waves without interpeak interval alterations and a lowering of the minimum response level. As for retrocochlear alteration, we considered an increase in wave absolute latency time and/or wave amplitude alteration. Following that, we assessed: the distribution of conductive/cochlear and retrocochlear findings, in agreement with gender and age range, absolute latencies associated with gender and the incidence of alterations in interpeak intervals. In children younger than twelve years of age and in the presence of risk factors we analyzed the incidence of conductive, cochlear and retrocochlear alterations, in accordance with the aforementioned criteria. The neonatal risk factors considered in our paper were the same ones described in the 2000 consensus from the “Joint Committee on Infant Hearing”^9^ (which include the American Academies of Audiology, Otolaryngology and Head and Neck Surgery, Pediatrics, Speech, Language and Hearing and the Education Council for the Hearing Impaired), to say:

1) less than 1500 grams of birth weight or preterm less than 34 weeks;
2) newborn small for the gestational age;
3) severe Perinatal asphyxia;
4) intracranial hemorrhage or leucoencephalomalacia;
5) suspected or confirmed congenital infection;
6) bacterial meningitis and/or septicemia;
7) use of ototoxic drugs;
8) hyperbilirubinemia;
9) family history of hearing loss;
10) parents who are related;
11) syndromes associated with hearing loss;
12) head and neck malformations;
13) Newborns who required neonatal ICU for 48 hours or more. All the data collected was stored in a database created in Access® and was later on analyzed and plotted in tables and charts.

## RESULTS

 

## DISCUSSION

**Graph 1:**
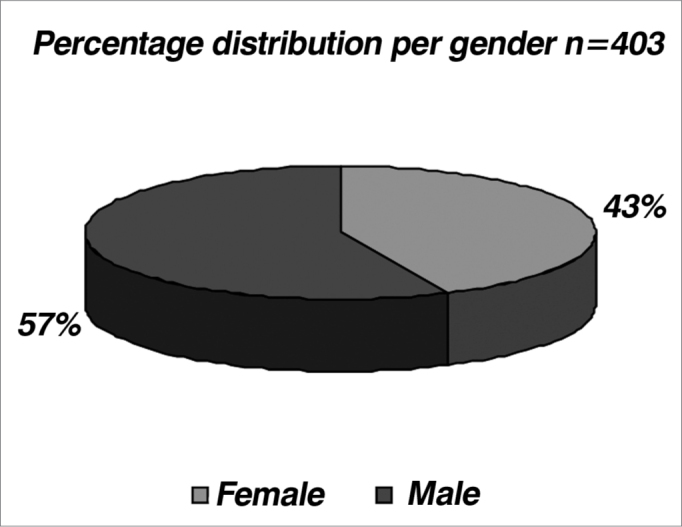
Distribution of the population studied by gender.

Within the analyzed period, we carried out brain stem audiometry (ABR) in 403 patients, with a predominance of males (57% - Table 1 and Graph 1).

**Table 1:** Population studied broken down by gender.

Gender	Females	Males	Total
n	173	230	403

**Graph 2:**
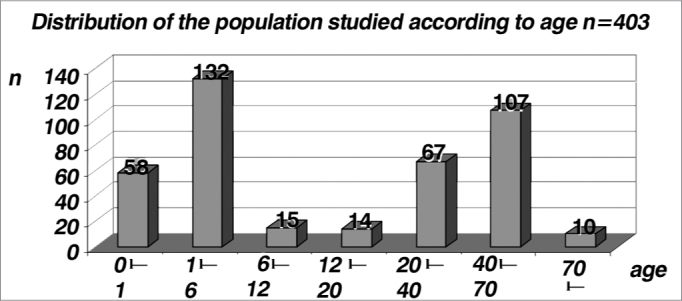
Distribution of the population studied by age, in years (n=403).

**Graph 3:**
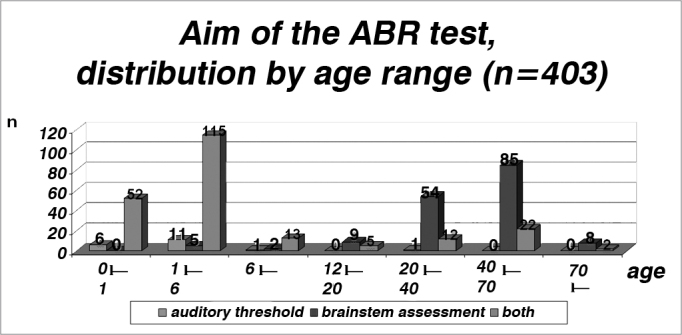
Reason for ordering the test (ABR), broken down by age range (n = 403).

As far as age is concerned, we observed two peaks of incidence:

1) age range between 1 and 6 years, having seen that in the pediatric population, classic tonal audiometry is more properly carried out within the mental ages of 5 and 7 years, and that is why we preferred the electrophysiological test;

2) age range between 40 and 70 years, since in it ABR is frequently used for the topodiagnosis of hearing loss or hearing complaints10 (Table 2 and Graph 2). Such fact is reinforced by Table 3 and Graph 3 which show a proper indication of ABR orders, in other words, in the pediatric range until 6 years, ABR prevailed in order to assess the auditory threshold in relation to the brain stem evaluation, while in adults the opposite happened. In the general distribution, 5% (19) of the tests were ordered with the aim of checking the auditory threshold, 40% (163) for stem assessment and 55% (221) for both.

**Table 2:** Distribution of the population studied by age.

Age (years)	n
0 l– 1	58
1 l– 6	132
6 l– 12	15
12 l– 20	14
20 l– 40	67
40 l– 70	107
70 l–	10

Total	403

**Table 3:** Reason for ordering the test (ABR), distributed by age range.

	Reason			
Age (years)	Hearing threshold	Brainstem assessment	both	Total
0 l– 1	6	0	52	58
1 l– 6	11	5	115	132
6 l– 12	1	2	13	15
12 l– 20	0	9	5	14
20 l– 40	1	54	12	67
40 l– 70	0	85	22	107
70 l–	0	8	2	10

Total	19	163	221	403

**Graph 4:**
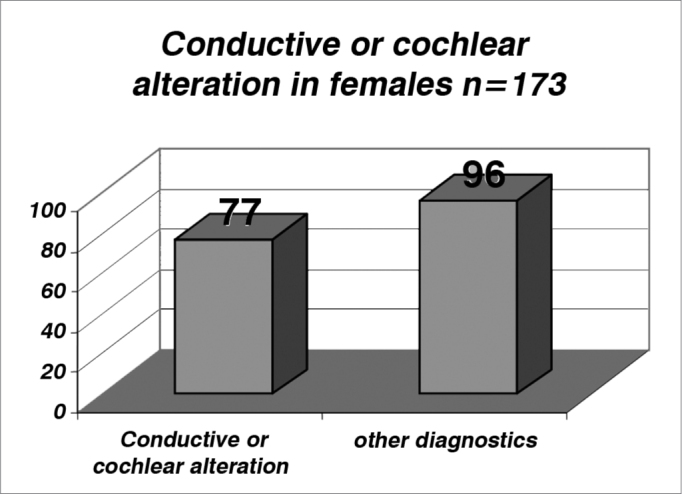
Incidence of female patients with conductive or cochlear alterations (index of 0.445 or 44.5%).

**Graph 5:**
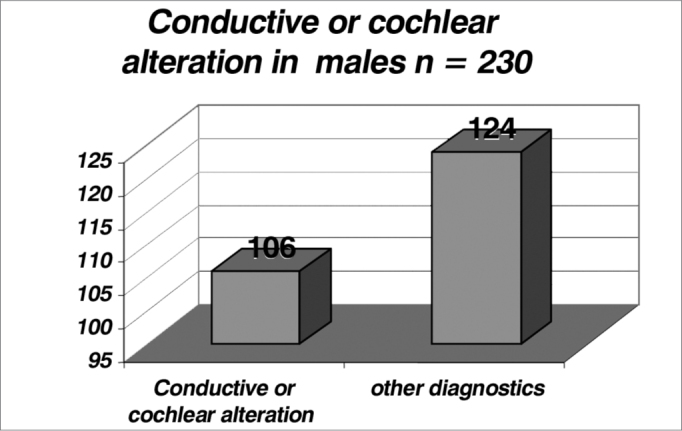
Incidence of male patients with conductive or cochlear alterations (index of 0.46 or 46%).

**Graph 6:**
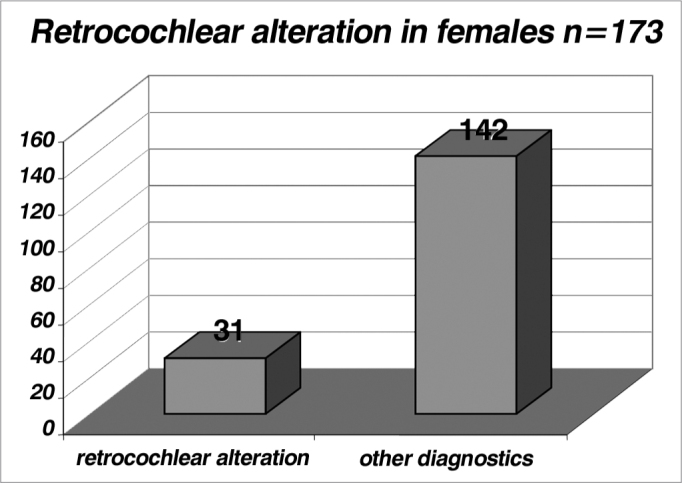
Incidence of female patients with retrocochlear alterations (index of 0.179 or 17.9%).

**Graph 7:**
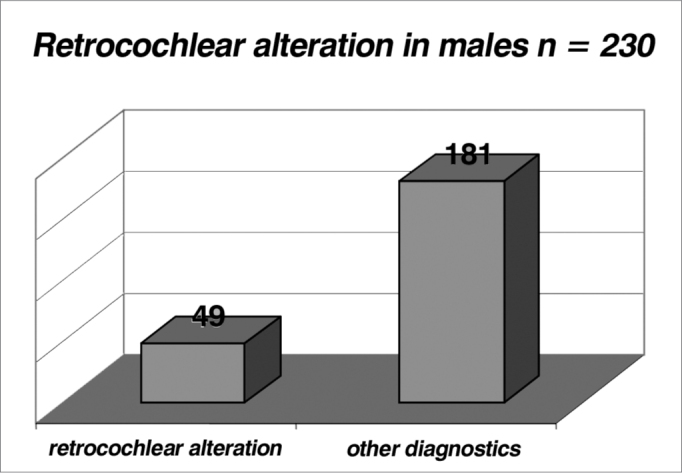
Incidence of male patients with retrocochlear alterations (index of 0.213 or 21.3%).

By analyzing the Table 4, Table 5 and Graphs 4 and 5, we noticed an incidence of conductive or cochlear alterations of 44.5% in females and of 46% in males, while the incidence of retrocochlear findings was of 17.9% in females and of 21.3% in males, the latter was statistically significant (p>0.05- Table 6, Table 7 and Graphs 6 and 7).

**Table 4:** Number of female patients with conductive or cochlear alterations.

Cochlear or conductive alteration	Other diagnosis	total
77	96	173

**Table 5:** Incidence of male patients with conductive or cochlear hearing loss.

Cochlear or conductive alteration	Other diagnosis	total
106	124	230

**Table 6:** Incidence of female patients with retrocochlear alterations.

Retrocochlear alteration	Other diagnosis	total
31	142	173

**Table 7:** Incidence of male patients with retrocochlear alterations..

Retrocochlear alteration	Other diagnostics	total
49	181	230

**Graph 8:**
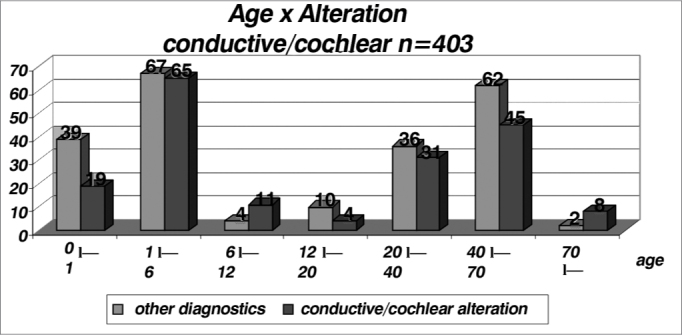
Distribution of patients with conductive or sensorineural alterations by age range in relation to other diagnostics (n = 403).

By analyzing the conductive or cochlear alterations by age range, we noticed a higher incidence in the age range of 70 years with a rate of 0.80 (very likely because of a hearing loss associated with aging and degenerative alterations)^11^. The general index was 0.45 (Table 8 and Graph 8).

**Table 8:** Distribution of patients with conductive or cochlear hearing loss per age range in relation to other diagnostics.

Age	Other diagnostics	Conductive/cochlear alterations	Total	Index
0 l-- 1	39	19	58	0,33
1 l-- 6	67	65	132	0,49
6 l-- 12	4	11	15	0,73
12 l-- 20	10	4	14	0,29
20 l-- 40	36	31	67	0,46
40 l-- 70	62	45	107	0,42
70 l--	2	8	10	0,8

Total	220	183	403	0,45

**Graph 9:**
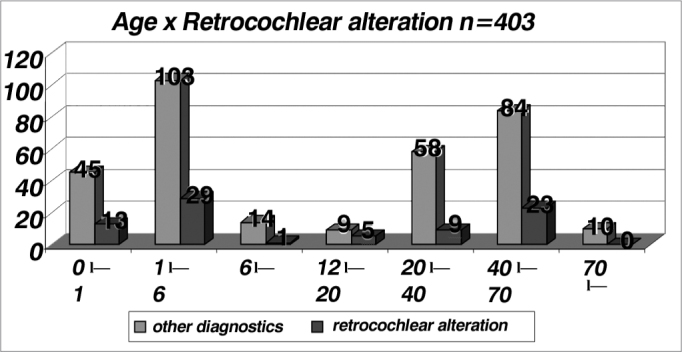
Distribution of patients with retrocochlear alterations per age range in relation to other diagnostics (n = 403).

As far as retrocochlear alterations are concerned we see a higher incidence in the age range between 12 and 20 years (n = 14), with an index of 0.36; and a general index of 0.20 (Table 9 and Graph 9), and when we analyze those children below one year of age (n= 58) the incidence of conductive/cochlear and retrocochlear alterations, even with the risk factors reported in the Method, we noticed indices of 0.33 and 0.22 respectively. These values are near and one of them is even lower than the one found in the general sample (n = 403), which were 0.45 and 0.20 respectively. Thus, we noticed that children with risk factors did not show a higher incidence of conductive, cochlear or retrocochlear alterations when compared to the general population studied.

**Table 9:** Distribution of the patients with retrocochlear alterations per age range in relation to other diagnosis (n = 403).

Age	Other diagnosis	Retrocochlear alterations	Total	Index
0 l-1	45	13	58	0,22
1 l-6	103	29	132	0,22
6 l-12	14	1	15	0,07
12 l-20	9	5	14	0,36
20 l-40	58	9	67	0,13
40 l-70	84	23	107	0,21
70 l--	10	0	10	0

Total	323	80	403	0,20

**Graph 10:**
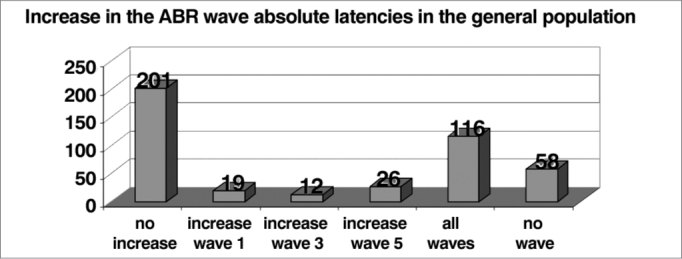
Increase in ABR wave absolute latency in the general population (n = 403).

In relation to the absolute latencies of waves I, III and V, we observed a difference between the genders, and they were higher among males, and this is in agreement with literature reports^12^(Table 10 and Graph 10).

**Table 10:** Increase in the absolute wave latency of the general population (n = 403).

Gender	No increase	Increase wave 1	Increase wave 3	Increase wave 5	Increase in all waves	No waves	Total
M	108	9	7	18	72	33	247
F	93	10	5	8	44	25	185

Total	201	19	12	26	116	58	432*

**Graph 11:**
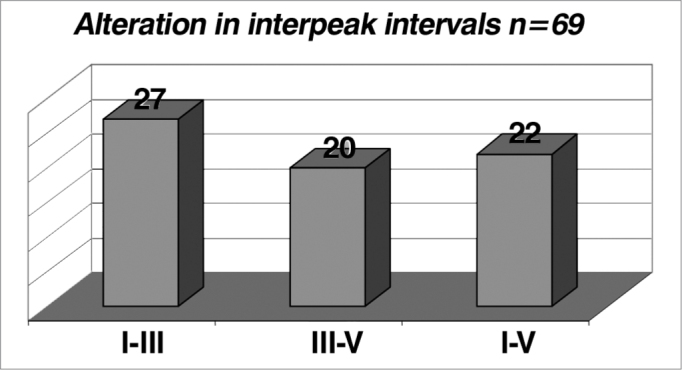
alterations in interpeak intervals (n = 69).

**Graph 12:**
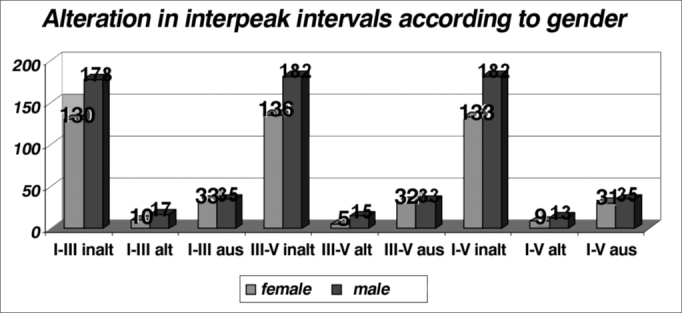
Alteration in interpeak intervals according to gender in the general population (n=403).

In relation to interpeak interval alterations (n = 69), we noticed that there were no differences between the genders, and the I-III interval was the one that presented the higher rate of alterations (39%)(Graph 11). Although the incidence of alterations in the interpeak intervals is higher for men (Table 11 and Graph 12), we highlight the greater incidence in males (230 men: 173 women studied) and small percentage differences. According to the literature, gender and age, the frequency of the stimulus and click phase impact on the absolute latencies, being however, controversial the influence of these factors in interpeak intervals^12^.

**Table 11:** Incidence of interpeak interval alterations broken down by gender. Males prevailed.

Gender	I-III unaltered	I-III altered	I-III absent	III-V unaltered	III-V altered	III-V absent	I-V unaltered	I-V altered	I-V absent	Total
F	130	10	33	136	5	32	133	9	31	173
M	178	17	35	182	15	33	182	13	35	230
Total	308	27	68	318	20	65	315	22	66	403

## CONCLUSIONS

1. Brain Stem Audiometry (ABR) is a useful and objective test to determine the electrophysiological threshold determination, especially in children.
2. In adults, the brain stem auditory pathways evaluation for topodiagnosis is more prevalent.
3. The prevalence of conductive and/or cochlear findings was much higher than that of retrocochlear disorder.
4. There is significant difference in relation to the retrocochlear electrophysiological findings as far as gender is concerned, and it is more pronounced in males.
5. Children below 1 year of age bearing risk factors did not present higher incidences of cochlear, conductive and retrocochlear alterations in the ABR when compared to the general population.
6. The waves’ absolute latencies were higher in males.
7. As to interpeak intervals, there was no gender difference and the I-III interval presented the higher incidence of alterations.
